# Prevalence and Social Risk Factors for Hearing Impairment in Chinese Children—A National Survey

**DOI:** 10.3390/ijerph14010088

**Published:** 2017-01-18

**Authors:** Chunfeng Yun, Zhenjie Wang, Jiamin Gao, Ping He, Chao Guo, Gong Chen, Xiaoying Zheng

**Affiliations:** Institute of Population Research, Peking University, Beijing 100871, China; chunfengyun@pku.edu.cn (C.Y.); zhenjie.wang@pku.edu.cn (Z.W.); gaojmin@pku.edu.cn (J.G.); pkuheping@pku.edu.cn (P.H.); chaoguo@pku.edu.cn (C.G.); chengong@pku.edu.cn (G.C.)

**Keywords:** hearing impairment, children, risk factor, China

## Abstract

Hearing impairment may affect children’s communication skills, social development, and educational achievement. Little is known about the prevalence of hearing impairment among Chinese children. Data were taken from the 2006 second China National Survey on Disability (CNSD). Hearing impairment was defined as moderate (41–60 dB HL), severe (61–80 dB HL), profound (81–90 dB HL), or complete (>91 dB HL). Logistic regression was used to estimate the odds ratio (OR) and 95% confidence intervals (CI). A weighted number of 567,915 hearing impairment children were identified, yielding a prevalence of 17.49 per 10,000 people (95% CI: 16.90–18.08), with prevention or treatment options possible for 64.6% of hearing impairment children. The main causes of hearing impairment were hereditary, tympanitis, and drug intoxication. Illiteracy in one or both parents (mother: OR = 1.388, 95% CI: 1.125–1.714, *p* < 0.0001; father: OR = 1.537, 95% CI: 1.152–2.049, *p* < 0.0001 relative to no school or primary school), annual family income lower than national average (OR = 1.323, 95% CI: 1.044–1.675, *p* = 0.0203, relative to higher than national average), household size larger than three people (OR = 1.432, 95% CI: 1.164–1.762, *p* = 0.0007, relative to smaller than three people) and single-mother family (OR = 2.056, 95% CI: 1.390–3.042, *p* = 0.0176, relative to intact family) were the independence risk factors for hearing impairment among Chinese children. Lower annual family income, male children, larger household size, single-mother family, and lower levels of maternal and paternal education were independent risk factors for hearing impairment for Chinese children. Further studies on hearing impairment prevention and the relationship between parental social factors and the risk of hearing impairment are needed.

## 1. Introduction

Hearing impairment is a common sensory disorder, affecting 27.8 million individuals of all ages in China [1]. Hearing loss of greater than 20 dB is the second most common impairment globally, affecting 1.33 billion (1.26–1.40 billion) people all over the world [2]. According to a 2012 World Health Organization (WHO) estimate, across the world there are 16 million (12–26 million) children who have a hearing impairment >35 dB HL, and the global prevalence of hearing impairment >35 dB HL among children 5–14 years of age was estimated to be 1.4% (95% uncertainty interval 1.0%–2.2%) [3]. The prevalence of hearing impairment among children aged 5–14 years was highest in South Asia, sub-Saharan Africa, and Asia Pacific [3]. In considering Canadian children aged 6–19 years old, 7.7% were estimated to have some degree of hearing loss for one or more pure-tone average [4]. Among U.S. children aged 6–19 years old, 12.5% (approximately 5.2 million) were estimated to have noise-induced hearing threshold shifts (NITS) in one or both ears [5]. In Africa, the estimated prevalence for hearing impairment children was 7.7% (2.4%–21.3%) using a cut-off of 25 dB HL [6]. In many countries or areas in the world, hearing impairment is the most frequent disability occurring during childhood [5,7,8,9,10,11], affecting 1 to 3 per 1000 newborns in the well-infant population and 2 to 4 per 1000 in infants with risk indicators [12,13]. For young children, even slight hearing impairment (15–24 dB) may need special auditory and speech accommodations to facilitate living as normal children [14]. Mild hearing impairment in young children can have serious negative effects on speech and language development and can result in difficulties in social-emotional development and educational achievement [15].

The causes of high prevalence of hearing impairment among children are not well understood, though it is very common [5,7,8,9,10,11] and can have important social and educational implications [15,16,17]. Previous research studies have found some risk factors of hearing impairment for newborns, such as neonatal intensive care unit admission, parental consanguinity, syndromes, and postnatal infections [18]. While for teenagers, noise exposure such as music listening had been found to be an important risk factor [8,19].

Hearing impairment is an invisible and chronic disability. With prompt diagnostic evaluation through newborn hearing screening and proper treatments, several cases of young children’s hearing impairment can be prevented or reduced. However, little is known regarding the prevalence of hearing impairment among Chinese children or the causes of hearing impairment, two forms of crucial forms of information that are needed to develop intervention strategies. Parental educational level, family income, or other social factors may influence the opportunity for timely screening and treatment. In the current study, we estimated the prevalence and main causes of hearing impairment among Chinese children aged 0–17 years and explored the associations between the parental and social factors on risk of hearing impairment among Chinese children by using a national population sample.

## 2. Methods

### 2.1. Study Population

The present sample was derived from a subpopulation of participants with hearing impairment from the Second China National Sample Survey on Disability conducted from 1 April to 31 May 2006. This survey was a nationally representative study that had been approved by the Chinese State Council (Guo Ban Fa No. 73 (2004)). A stratified, multiphase, and cluster probability sampling design was used in the selection of participants. A total of 734 counties (cities or districts), 2980 towns (townships or streets), and 5964 communities were selected from 31 provinces, autonomous regions, and municipalities directly under the Central Government in China (except Taiwan, Hong Kong, and Macao). As a result, 2,526,145 persons in 771,797 households were investigated in this survey. All participants signed informed consent documents provided by the Chinese Government [20].

### 2.2. Interviewers and Interviewing Procedures

Over 6000 doctors, more than 20,000 interviewers and 50,000 survey assistants participated in this survey. Prior to the survey (before 25 March 2006), information about the number of households, population, suspected disabled people, and children aged 0–6 years in the sampling community was collected.

Household surveys were conducted. All selected households and family members of each household were investigated by trained interviewers. For subjects aged seven years or older, a screen scale of disabilities was conducted by trained interviewers, and those suspected of having a hearing impairment were then examined by audiologists following diagnostic manuals to confirm a final diagnosis, if any, and to confirm its original causes. Children aged 0–6 years were first assessed by doctors in various specialties, and those suspected of having a hearing impairment were then examined by audiologists to confirm a final diagnosis of disability as well as the causes of disability. An appointment was made for a second visit if any children were not at home during the interview. If the child was again unavailable on the subsequent visit, information on that person was obtained from the other family members.

### 2.3. Identification of Children with Hearing Impairment

The definition and classification of hearing impairment were established by the Expert Committee of the Second China National Sample Survey on Disability based on the WHO International Classification of Functioning, Disability, and Health (WHO-ICF) [21]. Hearing impairment was diagnosed by the audiologists according to the manual that had previously been established by many medical experts. In agreement with this medical manual, the term *hearing impairment* was used to refer to permanent hearing impairment of varying degrees from any cause, or the inability to hear at all or to hear clearly any nearby sound or voice that negatively impact his or her daily life and participation in social activities [20]. For children over three years old, pure tone audiometry (at 500, 1000, 2000, and 4000 Hz) data were collected. The definition of hearing impairment for children over three years old used the cut-off value of 40 dB HL, which is in line with the definition of “disabling hearing impairment” given by the World Health Organization [22]. Similar to the grades of hearing impairment that were defined by WHO [22], hearing impairment was graded into moderate (41–60 dB), severe (61–80 dB), profound (81–90 dB), and complete (>91 dB) in this study. For children aged 0–2.9 years, free-field behavior audiometry data (at 1000, 2000, and 4000 Hz) was collected. Children aged 0–5 months were not included in this study. For children aged 6–12 months, a loudspeaker issued a test warble tone of 80 dB HL, and only profound (81–90 dB) and complete (>91 dB) hearing impairments were recorded. For children aged 13–36 months, a loudspeaker issued a test warble tone of 60 dB, and severe (61–80 dB), profound (81–90 dB), and complete (>91 dB) hearing impairments were recorded.

### 2.4. Data Analysis

We further categorized age groups (0–2.9, 3–5.9, or 6–17.9), gender (male or female), residence (rural areas or urban areas), regions (west, central, or east), annual family income divided by the number of persons in the household (national average or higher), household size (more than three people in a family, or three people or less in a family), mother’s or father’s education levels (illiterate, no school or primary school, junior middle school, senior middle school, college or more, unknown), family structure (intact family 1, intact family 2, step family, single-mother family, single-father family, or unknown). An unknown classification for the mother’s education levels means that the mother could not be found in our database. Similarly, an unknown in the father’s education levels means that the father could not be found in our database. Intact family 1 refers to children who lived with two biological parents. Intact family 2 refers to children who lived with one biological parent, while the other parent was not at home when the survey data was collected. The step family classification indicated that there was at least one step parent in the family. An unknown classification for the family structure means that both parents could not be found in our database.

We used standard weighting procedures to construct sample weights allowing for the complex survey sample design [23]. Variance and corresponding 95% confidence interval (CIs) were estimated by the Taylor series linearization method [24]. The Chi-square test was used to compare prevalence between different groups.

Two multivariable logistic regression models were used to calculate the adjusted ORs and 95% CIs. As different audiometry parameters were used in different age groups of children, we adjusted for the age group of the children in both of the two logistic regression models. In model 1, we focused on analyzing parental educational level on the risk of hearing impairment for the offspring and included the following confounders: child’s gender (male vs. female), residence of the child (rural vs. urban), annual family income (>national average vs. ≤national average), household size (>3 people vs. ≤3 people), maternal education level (illiterate, no school or primary school, middle school or more), paternal education level (illiterate, no school or primary school, middle school or more), and family structure (two-parent family vs. single-parent family). In model 2, we focused on family structure and the risk of hearing impairment in children and included the following confounders: child’s gender (male vs. female), residence of the child (rural vs. urban), annual family income (>national average vs. ≤national average), household size (>3 people vs. ≤3 people), and family structure (intact family 1, intact family 2, step family, single-father family, single-mother family, unknown). The procedures SURVEYFREQ and SURVEYLOGISTIC in the program SAS 9.1 (SAS Institute Inc., Cary, NC, USA) were utilized to perform data analyses [25]. We set a *p* value less than 0.05 as statistically significant.

## 3. Results

### 3.1. Prevalence of Hearing Impairment among Chinese Children

A total of 616,940 children aged 0–17 years were investigated in this survey. Above all, there were 9852 children with a disability of any type identified. Moreover, there were 1112 children with hearing impairment identified in this survey. By the standard weighting procedures, the population-weighted number of children with hearing impairment was 567,915, yielding a weighted prevalence of 17.49 per 10,000 people (95% CI: 16.90–18.08). Children aged 6–17 had a significantly higher rate than children aged 0–2 and 3–6 (*p* < 0.0001). There was a significant difference in the prevalence for children in rural and urban areas (18.88 vs. 13.44 per 10,000 people, *p* < 0.0001). Children living in western regions had a significantly higher rate than children living in eastern and central areas (*p* < 0.0001). Children with lower education level (illiteracy or primary school) parents had a significantly higher hearing impairment rate than children having higher education level (senior middle school or more) parents (*p* < 0.0001). Children from single-mother families and single-father families had much higher rates than children from intact families and step families (*p* = 0.0002). There was also a significant difference in the prevalence for children from lower and higher income families (18.83 vs. 11.30 per 10,000 people, respectively; *p* < 0.0001). Additional details are presented in Table 1.

### 3.2. The Main Cause and Combined Disabilities for Chinese Hearing Impairment Children

The main causes for hearing impairment of the 1112 Chinese children identified in this study were hereditary, tympanitis, and drug intoxication, and it was found that 64.6% of the hearing impairment cases could be prevented or treated (detail see in Table 2). The proportion for moderate (41–60 dB), severe (61–80 dB), profound (81–90 dB), and complete (>  91 dB) hearing impairment were 47.2%, 13.3%, 20.2%, and 19.2%, respectively. In various age groups, prevalence rates were different though in the same degree of hearing impairment (for further details see Table 3). Of all the 1112 hearing impairment children, 38.1% had hearing impairment only, 56.5% had a hearing impairment combined with a speech disability, 4.1% had a hearing impairment combined with an intelligence disability, 0.64% had a hearing impairment combined with physical disabilities, 0.39% had a hearing impairment combined with a vision disability, and 0.26% had a hearing impairment combined with a mental disability.

### 3.3. Socioeconomic Factors Associated with Hearing Impairment among Chinese Children

A logistic regression model was used to explore the parental social factors associated with the risk of hearing impairment. Because some of the children’s parents could not be found in the period of this survey, 118,336 healthy children and 261 hearing impairment children were removed from the logistic regression model. As such, we retained 851 hearing impairment children and 497,492 healthy children in the logistic analysis, and found the following factors were associated with hearing impairment among Chinese children: maternal education level (illiteracy: OR = 1.388, 95% CI: 1.125–1.714, *p* < 0.0001 relative to no school or primary school), paternal education level (illiteracy: OR = 1.537, 95% CI: 1.152–2.049, *p* < 0.0001 relative to no school or primary school), gender of the child (male OR = 1.210, 95% CI: 1.046–1.399, *p* = 0.0103 relative to female), annual family income (lower than national average OR = 1.323, 95% CI: 1.044–1.675, *p* = 0.0203, relative to higher than national average), and household size (larger than 3 people OR = 1.432, 95% CI: 1.164–1.762, *p* = 0.0007, relative to smaller than three people) (Table 4).

In the above logistic regression model, 118,597 children were not included. We used another logistic model which including the entire 616,940 children in our survey to analyze the family structure and other social factors on the risk of hearing impairment. The following factors were found to be associated with hearing impairment among Chinese children: living in rural areas (OR = 1.410, 95% CI: 1.178–1.687, *p* = 0.0002, relative to living in urban areas), annual family income lower than national average (OR = 1.681, 95% CI: 1.365–2.071, *p* < 0.0001, relative to higher than national average), household size larger than three people (OR = 1.256, 95% CI: 1.071–1.472, *p* = 0.0049, relative to smaller than three people), and coming from a single-mother family (OR = 2.056, 95% CI: 1.390–3.042, *p* = 0.0176, relative to intact family 1 that included two biological parents in home) (Table 5).

We compared the parent’s literacy level between hearing impairment children and healthy children in different family structures by using Chi square analysis, and found that the parents’ literacy level was significantly different between hearing impairment children and healthy children (single mother, *p* = 0.0153; single father, *p* = 0.0256; couple mother, *p* < 0.0001; couple father, *p* < 0.0001) (for further details see Table 6).

## 4. Discussion

This study revealed that the prevalence of hearing impairment among Chinese children aged 0–17 years old was 17.49 per 10,000 people, which was much lower than the prevalence of hearing impairment among children in Canada [4], the USA [5,26], and Africa [6]. The different cut-off values for hearing impairment in different surveys may be the main reason for the low prevalence of hearing impairment among Chinese children. In the Canadian survey, hearing impairment was defined as a pure-tone average >20 dB for 6- to 18-year olds and ≥26 dB for 19-year olds [4]. In the U.S. National Health and Nutrition Examination Survey, hearing impairment was defined as a pure-tone average >25 dB for 12–19 years olds [26] and Noise-Induced Hearing Threshold was defined as >16 dB HL for 6–19 years olds [5]. In Africa, the cut-offs for hearing impairment in children were usually 25 dB HL or 30 dB HL [6]. In our study, we used the cut-off 40 dB HL for hearing impairment.

The prevalence of hearing impairment among Chinese children aged 0–2.9 years was much lower than that in children ages 3–5.9 and 6–17.9 years. For the complete, severe, or moderate degree of hearing impairment, there were significant differences between the prevalence of 0–2.9, 3–5.9, and 6–17.9 year old children. In our study, the different interview and audiometry procedures between different age groups of children could have implications for the ascertainment of cases of hearing impairment. This may be an important reason for the differences in the prevalence rates by age group in the results. Furthermore, in December 2004, the Ministry of Health in P.R. China issued Technical Guidelines for the Screening of Neonatal Diseases which included the guideline of newborn hearing screening. The data used in this analysis was conducted in 2006, almost all of the children aged 0–2.9 years had received the public health services that were based on the hearing screening guidelines that were issued in the year 2004. The positive impacts of the prevention of hearing impairment for Chinese newborns may be another explanation for this result. Hearing impairment has the nature of being relatively hidden, and it may be especially uneasy to diagnose for little children. Former studies have showed that the prevalence of hearing impairment was high among older children [11] because of some unhealthy behaviors [10].

Our research indicated that both low maternal or paternal education levels appeared to be risk factors for offspring with hearing impairment. Additionally, we also found that lower annual family income was a risk factor for children with hearing impairment. In China, lower educational levels always accompany lower income. Previous research has showed that lower income was a risk factor for cytomegalovirus [27], which is the leading infectious agent causing congenital sensorineural hearing impairment [28]. A study using U.S. data also reported that children from families below the federal poverty threshold had significantly higher odds of hearing impairment than those above the threshold [10]. Parental education level may be related to informed decisions before or during pregnancy and child raising. A person with less education has a greater likelihood of being exposed to secondhand smoke [29] or working in hostile conditions [30], and such unhealthy lifestyle conditions may be harmful to offspring [31] and may contribute to congenital defects such as congenital sensorineural hearing impairment [28].

Single-mother families have a greater risk of having hearing impairment children. As a limitation of the survey design, we only have information regarding the family structure variable and we do not known whether children had hearing impairments before or after divorce for the single mother families. Therefore, according to our results, there may be two potential explanations. One is that having children with hearing impairments raises difficulties for single mothers to remarry. Another explanation is that single mother families have a higher risk for their children to experience unexpected hearing impairment. This result is similar with a previous study that single mothers with sole/main custody reported poorer self-rated health than couple mothers in intact families [32]. In the future, we need to study the reason for the higher prevalence of hearing impairment among children from single-mother families.

In this study, hearing impairment was more common among children from household size > three people than households with ≤ three people. One probable explanation is that the one child policy was used in China before this study date was collected. From the 1980s to 2006 when the data was collected, most of the urban population were only allowed to birth one child. Couples whose first child had some kind of disability were allowed to have another child. For people living in rural areas during the one child policy period, many families were able to have two or more children. However, public health care and perinatal care in rural areas was not as good as in urban areas, which may have led to a higher prevalence of hearing impairment in rural areas. In addition, the prevalence of other types of disability such as physical disability in rural areas are much higher than in urban areas [20]. The WHO also indicated that hearing impairment prevalence is highest in low- and middle-income regions [3]. This may be another explanation for higher prevalence of hearing impairment among children from household sizes larger than three people.

There were some limitations in this study. According to the WHO Media center suggestion, hearing impairment refers to a hearing loss greater than 30 dB HL in the better hearing ear in children [33]. In our study, the definition of hearing impairment was >40 dB HL. As such, some forms of slight hearing impairment were not included in our study. The different definition of hearing impairment may have resulted in the underestimation of the prevalence of hearing impairment among children in China. The data used in this study was collected in 2006. The public health problem may be changing on account of the rapid development in China since 2006. However, to the best of our knowledge, the data used in our study was the latest national survey data on children hearing impairment in China, and this was the first original English paper on the prevalence of hearing impairment among children in mainland China. In our next survey on hearing impairment, we will pay more attention to the prevalence of slight hearing impairment among Chinese children, and we will try to collect more information on conductive or sensory-hearing impairment, congenital or acquired hearing impairment, and the laterality of the hearing impairment. We believe that more studies on the prevention of hearing impairment in children is needed in the future.

## 5. Conclusions

Our study is the first to report on hearing impairment among Chinese children. The prevalence of hearing impairment was 17.49 per 10,000 Chinese children aged 0–17 years. Independent risk factors include income lower than national average, household size larger than three people, single-mother families, lower levels of parental education. Greater public attention to the hearing impairment of offspring is required among those with less education, less income, larger household sizes, and single-mother families.

## Figures and Tables

**Table 1:** Prevalence rate of hearing impairment among Chinese children (per 10,000 people).

Variable	Survey Participants	Hearing Impairment Cases			*p-*Value
	*n*	*n*	Weighted *n*	Weighted Rate	
Total	616,940	1112	567,915	17.49 (16.90–18.08)	
Children Age (year)					<0.0001
0–2.9	82,705	76	40,250	9.25 (8.09–10.41)	
3–5.9	84,040	135	71,867	16.25 (14.72–17.78)	
6–17.9	450,825	901	455,798	19.23 (18.51–19.95)	
Child’s Gender					0.1492
Male	330,053	612	317,999	18.23 (17.42–19.04)	
Female	286,887	500	249,916	16.63 (15.82–17.44)	
Residence					<0.0001
Rural	449,500	898	455,806	18.88 (18.18–19.58)	
Urban	167,440	214	112,109	13.44 (12.38–14.50)	
Region					<0.0001
East	207,031	305	174,286	15.10 (14.11–16.09)	
Central	188,747	282	165,253	15.19 (14.26–16.12)	
West	221,162	525	228,376	22.71 (21.58–23.84)	
Annual family income					<0.0001
≤National average	507,773	992	502,406	18.83 (18.16–19.50)	
>National average	109,167	120	65,509	11.30 (10.18–12.42)	
Household size					0.0011
≤3 people	432,505	849	427,612	18.67 (17.94–19.40)	
>3 people	184,435	263	140,303	14.65 (13.71–15.59)	
Mother’s educational level					<0.0001
Illiteracy	62,370	227	101,892	33.83 (31.23–36.43)	
No school or primary school ^#^	186,383	379	201,070	20.02 (18.9–21.14)	
Junior middle school	211,338	265	141,459	12.42 (11.6–13.24)	
Senior middle school	53,255	48	22,998	8.77 (7.39–10.15)	
College or more	23,641	13	5693	5.25 (3.69–6.81)	
Unknown	79,953	180	94,802	21.90 (20.14–23.66)	
Father’s educational level					<0.0001
Illiteracy	17,754	91	36,199	46.40 (40.87–51.93)	
No school or primary school ^#^	143,320	345	173,124	23.21 (21.82–24.60)	
Junior middle school	254,562	364	194,211	14.14 (13.33–14.95)	
Senior middle school	72,884	79	41,010	11.03 (9.69–12.37)	
College or more	29,727	18	8406	5.93 (4.41–7.45)	
Unknown	98,693	215	114,965	21.41 (19.88–22.94)	
Family structure *					0.0002
Intact family 1	479,308	810	410,380	16.34 (15.70–16.98)	
Intact family 2	39,333	67	37,790	17.32 (15.15–19.49)	
Step family	19,457	42	20,711	21.56 (18.02–25.10)	
Single-mother family	8677	31	13,487	32.76 (32.12–33.40)	
Single-father family	10,108	28	12,973	24.67 (19.62–29.72)	
Unknown	60,057	134	72,575	22.16 (20.13–24.19)	

CI: Confidence Interval. * Intact families 1 refers to children who lived with two biological parents. Intact families 2 refers to children who lived with one biological parent, while the other parent was not at home when the survey data was collected. The step family classification indicates that there was at least one step parent in the family. The unknown classification for the family structure means that both parents could not be found in our database. **^#^** In China, children enroll in primary school at the age of 6–8, and it takes 5–6 years to complete the primary school education.

**Table 2:** The main causes of hearing impairment for Chinese children (*n* = 1112).

Main Cause for Hearing Impairment	*n*	%	Potential Prevention Methods
Hereditary	198	17.8	Some kinds of hereditary hearing impairment can be prevented by assisted reproductive technologies and preconception health care services
Tympanitis	159	14.3	Quality children care services
Drug intoxication	138	12.4	Quality children care services
Infectious disease	47	4.2	Quality children care services
Pregnancy viral infection	65	5.8	Quality maternal care services
Asphyxia neonatorum	25	2.2	Quality newborn care services
Pregnancy general disease	21	1.9	Quality maternal care services
Premature birth and low body weight	19	1.7	Quality maternal care services
Hyperbilirubinemia	5	0.5	Quality newborn care services
Autoimmune disease and immunodeficiency disease	5	0.4	Quality children care services
Wound or accident	33	3	Health education
Noise and knocking	4	0.4	Health education
Undetermined	393	35.4	Unknown

**Table 3:** Prevalence of hearing impairment by age group and degree of hearing impairment (per 10,000 people).

Degree of Hearing Impairment	0–2.9 Years	3–5.9 Years	6–17.9 Years	*p*-Value
	*n* = 82,075	*n* = 84,040	*n* = 450,825	
Complete (>91 dB HL)	6.04 (5.14–6.94)	9.64 (8.51–10.77)	8.56 (8.08–9.04)	0.0389
Profound (81–90 dB HL)	1.62 (1.15–2.09)	2.26 (1.64–2.88)	2.61 (2.35–2.87)	0.3162
Severe (61–80 dB HL)	1.60 (1.14–2.06)	2.74 (2.13–3.35)	3.93 (3.60–4.16)	0.0041
Moderate (41–80 dB HL)	-	1.61 (1.08–2.14)	4.13 (3.81–4.55)	0.0041

**Table 4:** Parental social level on the risk of hearing impairment among Chinese children (hearing impairment vs. healthy children).

Variable	Odds Ratio (95% CI)	*p*-Value
Child’s Gender (Ref.: Female)		
Male	1.210 (1.046–1.399)	0.0103
Residence (Ref.: Urban)		
Rural	1.061 (0.857–1.315)	0.5851
Annual family income (Ref.: >National average)		
≤National average	1.323 (1.044–1.675)	0.0203
Household size (Ref.: ≤3 people)		
>3 people	1.432 (1.164–1.762)	0.0007
Maternal educational level (Ref.: No school or primary school)		
Illiteracy	1.388 (1.125–1.714)	<0.0001
Middle school or above	0.721 (0.598–0.870)	<0.0001
Paternal educational level (Ref.: No school or primary school)		
Illiteracy	1.537 (1.152–2.049)	<0.0001
Middle school or above	0.721 (0.607–0.855)	<0.0001
Family structure (Ref.: Two-parent family)		
Single-parent family	1.897 (0.266–13.542)	0.5230

Adjusted for children’s age group. Ref.: reference category.

**Table 5:** Family structure on the risk of hearing impairment among Chinese children (hearing impairment vs. healthy children).

Variable	Odds Ratio (95% CI)	*p*-Value
Child’s Gender (Ref.: Female)		
Male	1.128 (0.994–1.280)	0.0615
Residence (Ref.: Urban)		
Rural	1.410 (1.178–1.687)	0.0002
Annual family income (Ref.: >National average)		
≤National average	1.681 (1.365–2.071)	<0.0001
Household size (Ref.: ≤3 people)		
>3 people	1.256 (1.071–1.472)	0.0049
Family structure (Ref.: Intact family 1) *		
Intact family 2	1.177 (0.909–1.524)	0.2171
Step family	1.278 (0.919–1.777)	0.6436
Single-father family	1.473 (0.978–2.218)	0.6807
Single-mother family	2.056 (1.390–3.042)	0.0176
Unknown	1.442 (1.185–1.754)	0.5876

Adjusted for children’s age group. Ref.: reference category. * Intact families 1 refers to children who lived with two biological parents. Intact families 2 refers to children who lived with one biological parent, while the other parent was not at home when the survey data was collected. The step family classification indicates that there was at least one step parent in the family. The unknown classification for the family structure means that both parents could not be found in our database.

**Table 6:** Percentage of parental educational level by family structure (%).

Educational Level			*n*	Illiteracy	No School or Primary School	Junior Middle School	Senior Middle School	College or More	*p*-Value
Single-parent family	Single mother	Healthy	8646	18.2	29.7	30.4	14.5	7.2	0.0153
		Hearing impairment cases	31	41.9	22.6	25.8	6.5	3.2	
	Single father	Healthy	10,080	8.8	37.0	40.0	11.0	3.2	0.0256
		Hearing impairment cases	28	25.0	35.7	25.0	14.3	0.0	
Two-parent family *	Couple mother	Healthy	527,095	11.5	34.8	39.5	9.9	4.4	<0.0001
		Hearing impairment cases	900	23.8	41.3	28.4	5.1	1.3	
	Couple father	Healthy	507,028	3.3	27.5	49.3	14.1	5.8	<0.0001
		Hearing impairment cases	869	9.7	38.6	41.1	8.6	2.1	

* Single family included single-mother family and single-father families. Two-parent family included intact family 1, intact family 2, and step family.
